# Clinical features of patients with septic shock-triggered Takotsubo syndrome: a single-center 7 case series

**DOI:** 10.1186/s12872-022-02787-3

**Published:** 2022-07-29

**Authors:** Chengqiao Jing, Yan Wang, Chunmiao Kang, Daoran Dong, Yuan Zong

**Affiliations:** 1 Intensive Care Unit, Shaanxi Provincial People’s Hospital, No. 256, Youyi West Road, Beilin District, Xian, 710000 China; 2 Department of Ultrasound, Shaanxi Provincial People’s Hospital, Xian, China

**Keywords:** Takotsubo syndrome, Septic shock, Sepsis-induced myocardial dysfunction, Case series

## Abstract

**Background:**

Myocardial dysfunction is common in septic shock and has long been recognized. Takotsubo syndrome is an acute and usually reversible myocardial injury without evidence of an obstructive coronary artery disease, yet little is known about this syndrome in septic shock patients.

**Case presentation:**

Among 84 septic shock patients admitted to the ICU over a period of 8 months, 7 patients (8.3%) were diagnosed with Takotsubo syndrome. The percentage of men was 71%, and the mean age was 58 (19–87) years. Sudden hemodynamic deterioration and/or dyspnea were the presenting symptoms in 6 patients. T-wave inversion was the major ECG anomaly in 5 patients. The mean left ventricular ejection fraction was 31.8% (20.0–53.0). Mild elevation of cardiac troponin disproportionate to the extent of regional wall motion abnormalities was present in all patients. Cardiac complications occurred in 6 patients. The mean time to recover the cardiac function was 6.5 (3–11) days. In-hospital death was observed in 2 patients.

**Conclusions:**

Takotsubo syndrome is not uncommon in septic shock patients and may be the cause of some patients with sepsis-induced myocardial dysfunction. New-onset hemodynamic and respiratory worsening could arouse the suspicion of Takotsubo syndrome and prompt the screening for this syndrome using echocardiography in this clinical context.

## Introduction

Takotsubo syndrome (TTS) is a transient but severe myocardial dysfunction, which leads to acute heart failure with the absence of a significant epicardial coronary artery disease [1]. It was originally introduced by Sato et al. in 1990 and has been increasingly recognized. TTS is usually, but not always, related to a preceding stressful event (physical, emotional or combined), and it is more prevalent in postmenopausal women [2]. Since septic shock can be considered as a severe physical stress factor, it may potentially trigger TTS. Indeed, some cases of sepsis-induced TTS have been described during the last 30 years [3]. In this work, we present 7 cases of septic shock-induced TTS from our intensive care unit (ICU).

## Patients and methods

We prospectively investigated septic shock patients who were admitted to the ICU at Shaanxi Provincial People’s Hospital from January 2021 to August 2021. Transthoracic echocardiography (TTE) was performed within the first 24 h of ICU admission. Then, it was repeated daily and in case of respiratory or hemodynamic worsening to screen for TTS. Sepsis is defined as life-threatening organ dysfunction caused by an extreme, dysregulated host response to infection. Organ dysfunction can be identified by an increase in the Sequential Organ Failure Assessment (SOFA) score of ≥ 2 points consequent to the infection. Septic shock is a subset of sepsis, which is defined as persistent hypotension requiring vasopressors to maintain an mean arterial pressure of ≥ 65 mmHg with a serum lactate level of > 2 mmol/L despite adequate volume resuscitation [4]. TTS was diagnosed based on the new International Takotsubo Diagnostic Criteria (InterTAK Diagnostic Criteria) from the International Expert Consensus Document on Takotsubo Syndrome [5] according to the following criteria: (1) The patient shows transient left ventricular (LV) dysfunction (hypokinesia, akinesia, or dyskinesia) presenting as apical ballooning or midventricular, basal or focal wall motion abnormalities. Right ventricular (RV) involvement can be present. The regional wall motion abnormality usually extends beyond a single epicardial vascular distribution. However, in rare cases the regional wall motion abnormality can be present in the subtended myocardial territory of a single coronary artery (in this case it is called focal TTS). (2) New electrocardiogram (ECG) abnormalities are present (ST-segment elevation/depression, T-wave inversion and QTc prolongation). However, in rare cases no ECG changes could be observed. (3) The levels of cardiac biomarkers (troponin and creatine kinase) are moderately elevated in most cases. It is common to see a significant elevation of the brain natriuretic peptide (BNP). (4) A significant coronary artery disease is not a contraindication in Takotsubo syndrome. (5) The patient has no evidence of infectious myocarditis. The study was approved by the institutional review board, and all patients or relatives signed a general informed consent form.

## Case presentation

**Fig. 1:**
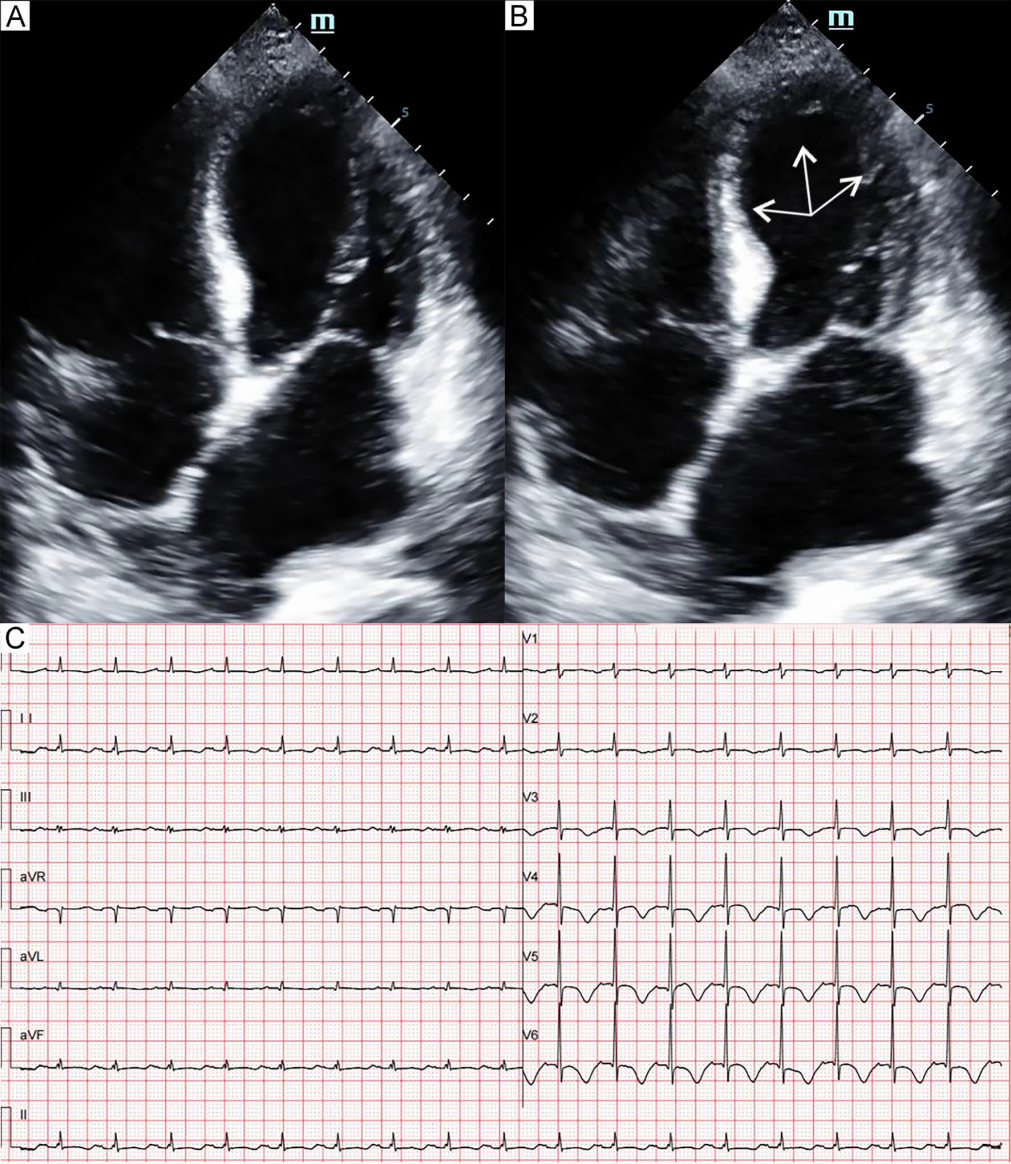
Patient 1. Apical four-chamber view of the echocardiography at end-diastole (**A**) and end-systole (**B**) demonstrated akinesia (arrows) of the apical and midventricular segments. Electrocardiogram (**C**) showed diffuse T-wave inversion and QTc prolongation

**Fig. 2:**
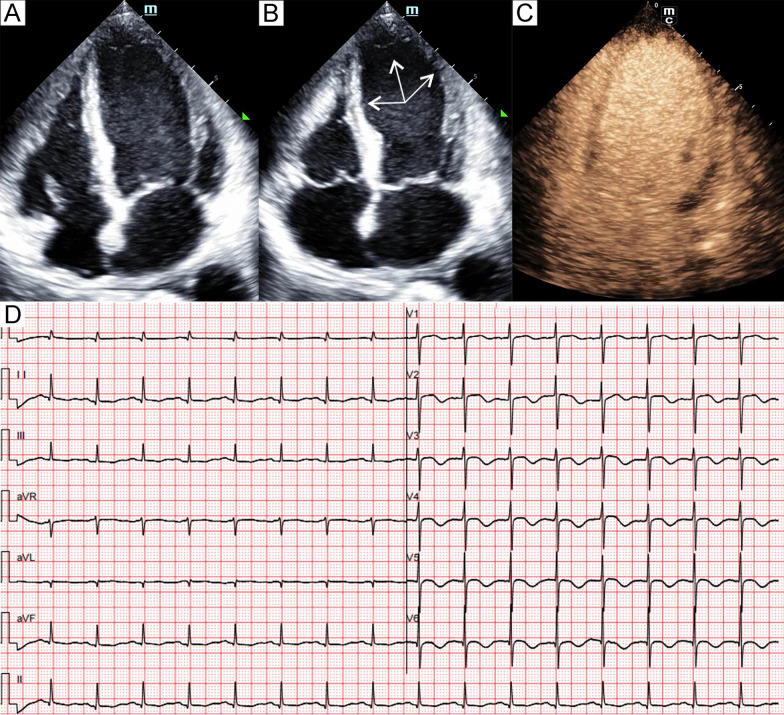
Patient 2. Apical four-chamber view of the echocardiography at end-diastole (**A**) and end-systole (**B**) demonstrated akinesia (arrows) of the apical and midventricular segments. Myocardial contrast echocardiography (**C**) showed homogeneous and equal enhancement intensity in the basal, midventricular and apical segments in the focused apical four-chamber view. Electrocardiogram (**D**) showed diffuse T-wave inversion and QTc prolongation

**Fig. 3:**
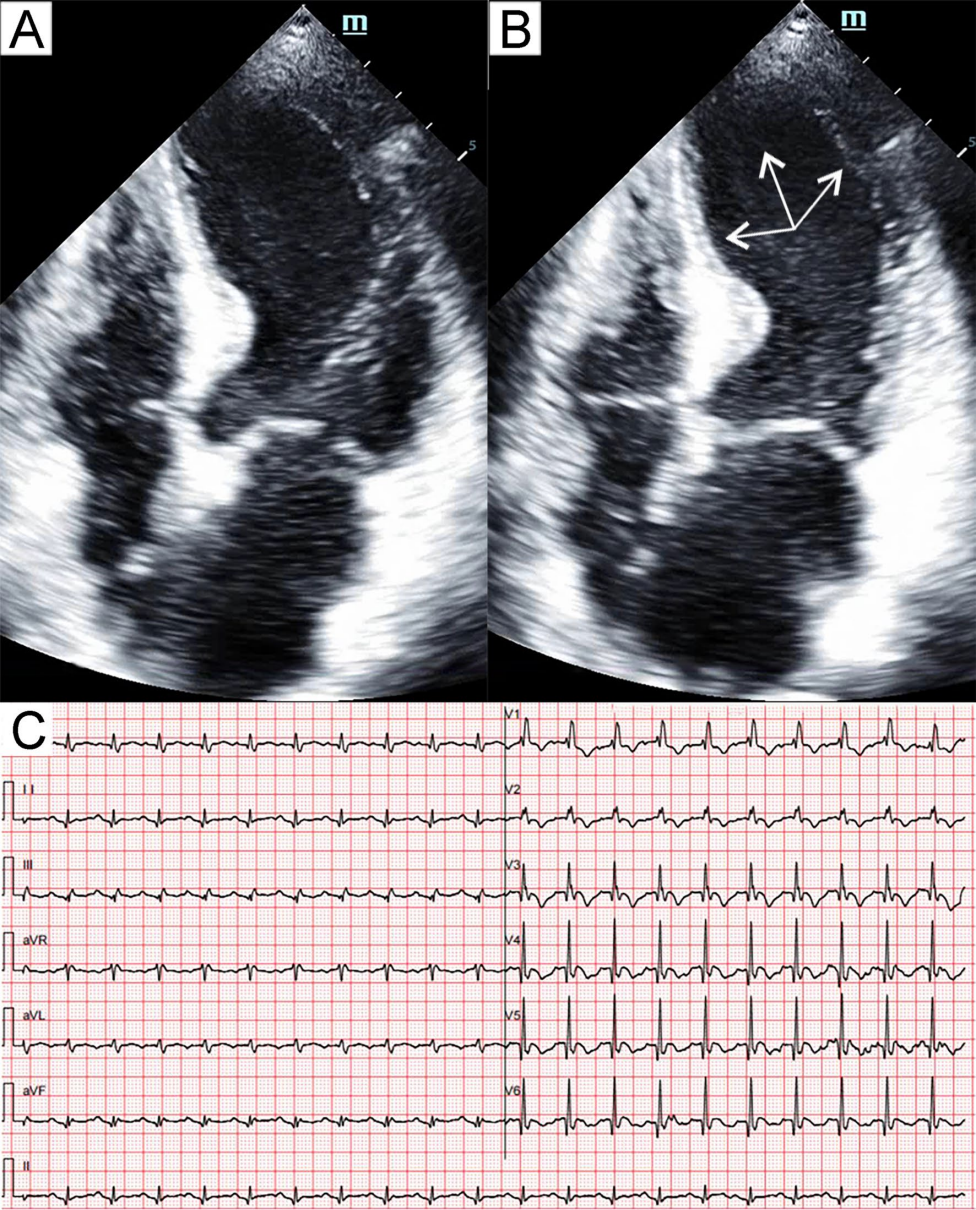
Patient 3. Apical four-chamber view of the echocardiography at end-diastole (**A**) and end-systole (**B**) demonstrated akinesia (arrows) of the apical and midventricular segments. Electrocardiogram (**C**) showed diffuse T-wave inversion

**Fig. 4:**
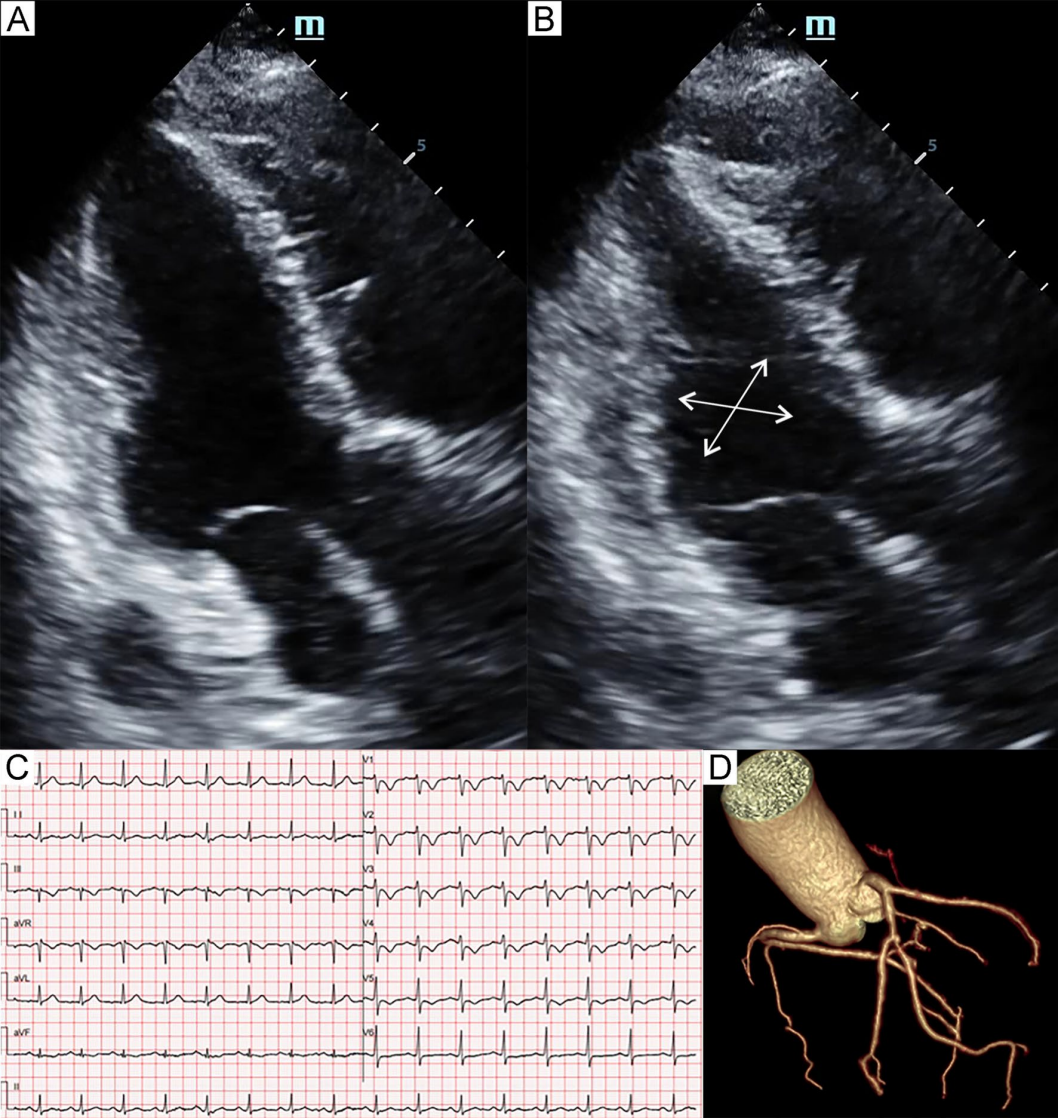
Patient 4. Apical long-axis view of the echocardiography at end-diastole (**A**) and end-systole (**B**) demonstrated severe hypokinesia (arrows) of the midventricular and basal segments. Electrocardiogram (**C**) showed diffuse T-wave inversion. Coronary computed tomography angiography (**D**) showed non-obstructive coronary artery disease

**Fig. 5:**
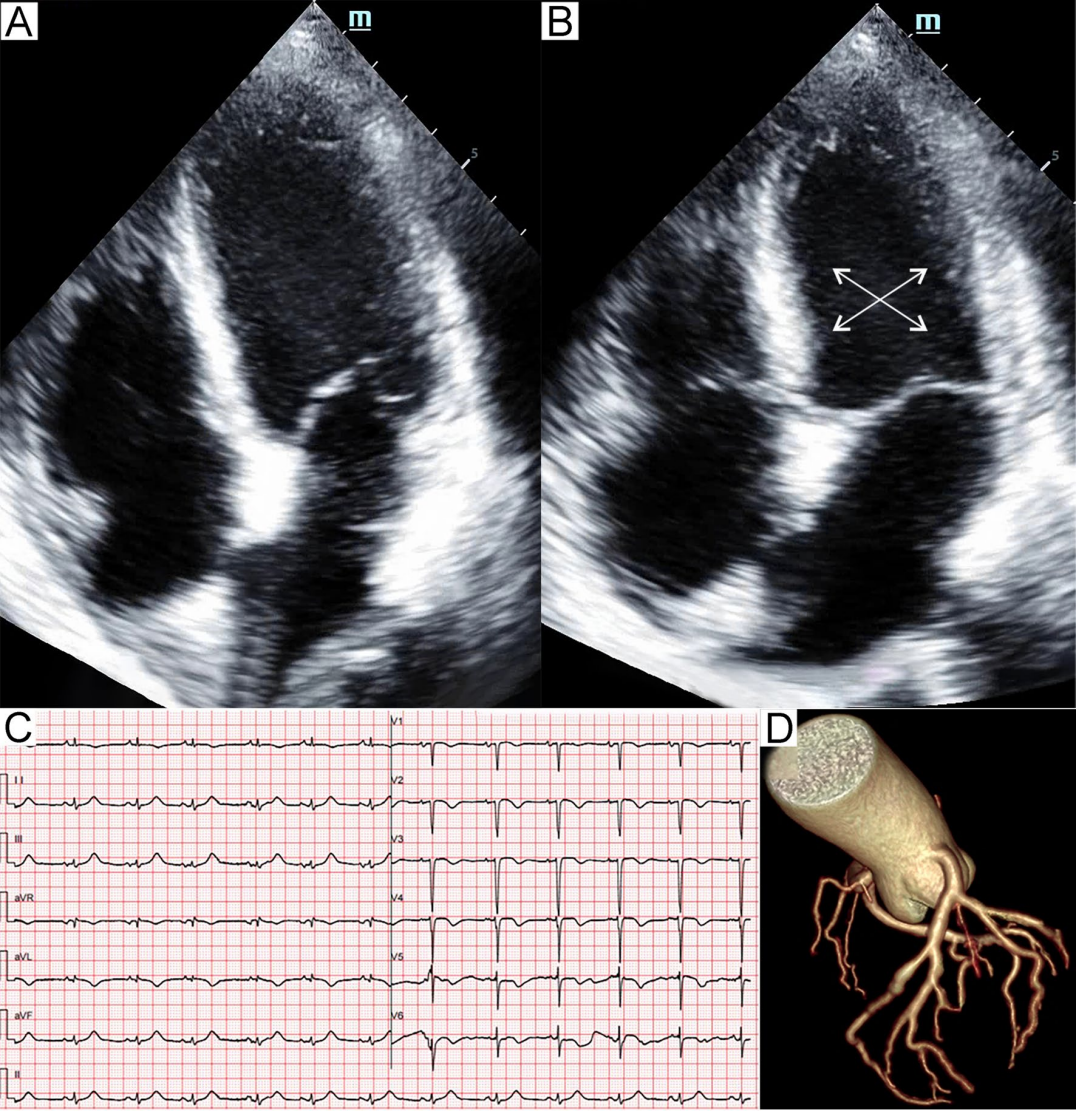
Patient 5. Apical four-chamber view of the echocardiography at end-diastole (**A**) and end-systole (**B**) demonstrated severe hypokinesia (arrows) of the midventricular and basal segments. Electrocardiogram (**C**) showed diffuse T-wave inversion. Coronary computed tomography angiography (**D**) showed a segment of left anterior descending artery (LAD) with myocardial bridging

**Fig. 6:**
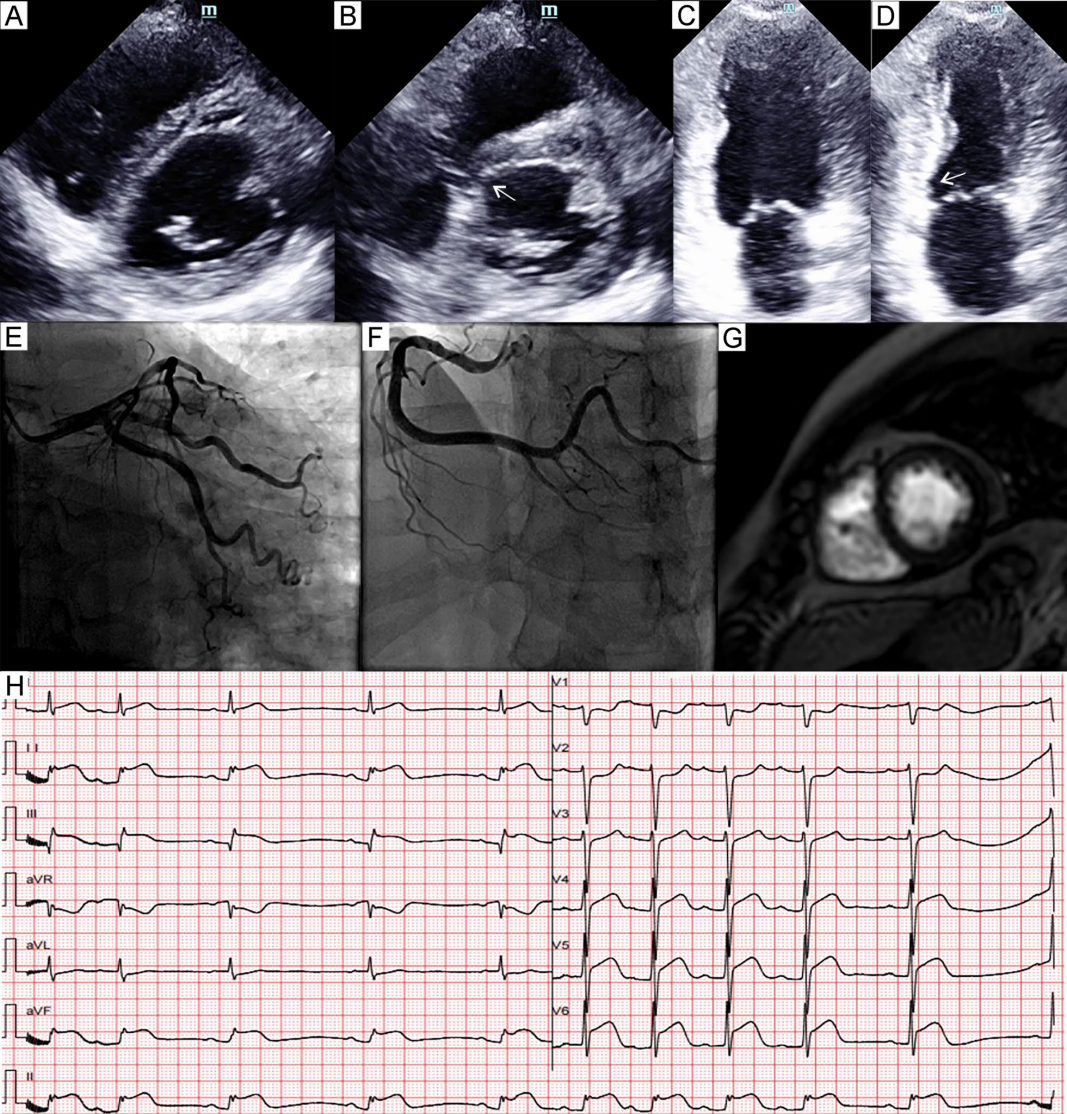
Patient 6. Parasternal short axis view of the echocardiography at end-diastole (**A**) and end-systole (**B**) demonstrated akinesia (arrows) of the basal segment of inferoseptal wall. Apical two-chamber view at end-diastole (**C**) and end-systole (**D**) demonstrated akinesia (arrows) of the basal segment of inferior wall. Coronary angiography (**E** and **F**) showed normal coronary arteries. Late gadolinium enhancement image of cardiac magnetic resonance (**G**) demonstrated the absence of significant necrosis or fibrosis. Electrocardiogram (**H**) showed ST-segment elevation in leads II, III, aVF, V5–V6 and QTc prolongation

**Fig. 7:**
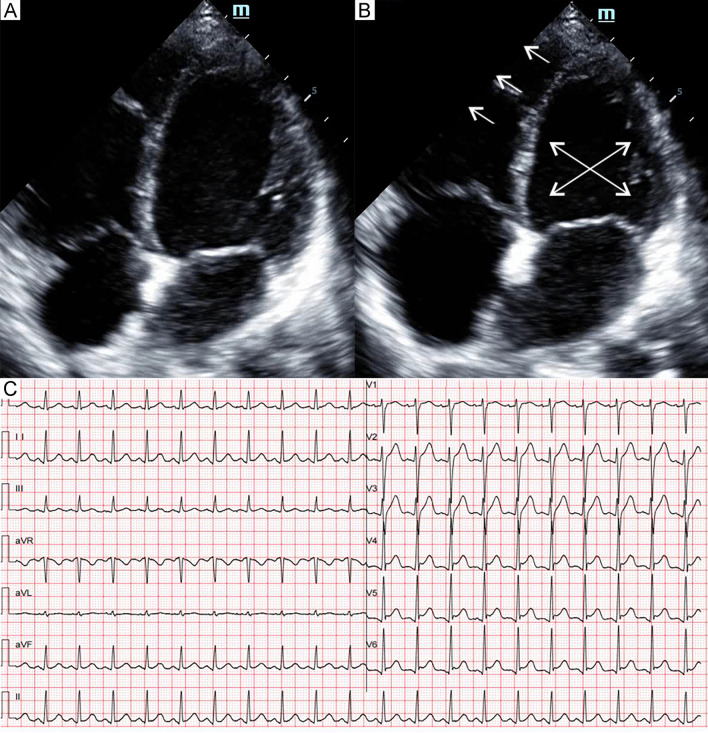
Patient 7. Apical four-chamber view of the echocardiography at end-diastole (**A**) and end-systole (**B**) demonstrated akinesia of the midventricular and basal segments of the left ventricle and right ventricular dysfunction (arrows). Electrocardiogram (**C**) showed mild ST-segment elevation in leads V4–V6

A total of 84 septic shock patients were admitted to our ICU during the investigation period, among which 7 patients were diagnosed with TTS (8.3%). The patients’ characteristics are listed in Table 1. The mean age was 58 years (19 to 87 years). Five patients (71%) were men. Only one patient had angina-like chest pain, while dyspnea and/or hemodynamic deterioration consisting of new tachycardia and aggravation of hypotension were the most common presenting symptoms in the remaining six patients. All the patients had an abnormal electrocardiogram with T-wave inversion (n = 5) or ST-segment elevation (n = 2). Transient prolongation of the corrected QT interval appeared in 3 patients. All the patients had extremely high BNP or N-terminal pro-brain natriuretic peptide (NT-proBNP) levels, with mild elevated troponin I (TnI) or troponin T (TnT). TTE showed a typical apical ballooning pattern of TTS in 3 patients (Figs. 1B, 2B, 3B), mid-basal (inverted) pattern in 2 patients (Figs. 4B, 5B), focal pattern in 1 patient (Fig. 6B, D), and biventricular pattern in 1 patient (Fig. 7B). Meanwhile, no left ventricular outflow tract obstruction (LVOTO) was observed. Imaging modalities were performed to rule out an acute coronary syndrome (ACS), including coronary angiography (CAG, patient 6), coronary computed tomography angiography (CCTA, patients 4 and 5) and myocardial contrast echocardiography (MCE, patient 2). Results are presented in Figs. 2, 4, 5, 6. CAG in patient 6 was normal. CCTA in patient 4 demonstrated 37% stenosis of the middle segment of the left anterior descending artery (LAD). CCTA in patient 5 revealed myocardial bridging of the mid-LAD. MCE in patient 2 showed no perfusion defects of the myocardium in the basal, middle or apical segments. In patient 6 (with a focal TTS), cardiac magnetic resonance (CMR) imaging was performed on day 9 and demonstrated a significant recovery of the segmental wall motion abnormality and the absence of late gadolinium-enhancement (LGE), which indicates myocardial infarction and myocarditis (Fig. 6G). These tests were not performed on the remaining 3 patients for the following reasons : patient 7 was a young man with a low suspicion of ACS and had a complete recovery of the cardiac function at discharge, patients 1 and 3 had a very poor general condition, were unable to undergo these examinations, and refused further workup after remarkable improvement of the LV systolic function.

**Table 1:** Characteristics of patients with septic shock-triggered Takotsubo syndrome

	Age (years)	Sex	Infection site	Microorganism	Presenting symptom	Cardiac biomarkers
1	87	M	Pressure ulcers	*Enterococcus faecium*	Dyspnea	TnI + (1.08 ng/mL)
					Hemodynamic instability	BNP + (2335 pg/mL)
2	67	F	Peritonitis	*Escherichia coli*	Hemodynamic instability	TnT + (0.159 ng/mL)
						NT-proBNP + (10,655 pg/mL)
3	63	M	CR-BSI	*Candida tropicalis*	Dyspnea	TnT + (0.447 ng/mL)
					Hemodynamic instability	NT-proBNP + (> 35,000 pg/mL)
4	56	F	Urosepsis	*Escherichia coli*	Dyspnea	TnI + (1.75 ng/mL)
					Hemodynamic instability	BNP + (2313 pg/mL)
5	63	M	CR-BSI	*Enterobacter cloacae* *Klebsiella oxytoca*	Dyspnea	TnT + (0.115 ng/mL)
						NT-proBNP + (13,943 pg/mL)
6	54	M	Cholangitis	Not clear	Chest pain	TnT + (0.273 ng/mL)
						NT-proBNP + (2222 pg/mL)
7	19	M	Wound	Not clear	Dyspnea	TnI + (2.11 ng/mL)
					Hemodynamic instability	BNP + (1798 pg/mL)

	ECG abnormalities			TTS pattern	LVEF (%)	Pulmonary oedema	Cardiogenic shock	Vasopressor/Inotrope	MV	HFNC	Recovery time (days)	Death
	ST elevation	T-wave inversion	QTc prolongation									
1	No	Diffuse	Yes	Typical	20	Yes	Yes	NE, MET Lev, Dob	Yes	No	6	Yes
2	No	Diffuse	Yes	Typical	20	Yes	Yes	NE, MET Lev, Dob	Yes	No	11	Yes
3	No	Diffuse	No	Typical	23	Yes	Yes	NE, MET Lev	No	Yes	10	No
4	No	Diffuse	No	Inverted	30	Yes	No	NE, DA Lev	No	No	4	No
5	No	Diffuse	No	Inverted	45	Yes	No	MET, DA Lev	No	Yes	3	No
6	II, III, aVF V5-V6	No	Yes	Focal	53	No	No	NE	No	No	9	No
7	V4-V6	No	No	Biventricular	32	Yes	No	NE, Lev	No	No	6	No

*BNP* brain natriuretic peptide, *CR-BSI* central venous catheter-related bloodstream infections, *DA* dopamine, *Dob* dobutamine, *ECG* electrocardiogram, *F* female, *HFNC* High-flow nasal cannula, *Lev* Levosimendan, *LVEF* left ventricular ejection fraction, *M* male, *MET* metaraminol, *MV* mechanical ventilation, *NE* norepinephrine, *NT-proBNP* N-terminal pro-brain natriuretic peptide, *TnI* troponin I, *TnT* troponin T, *TTS* Takotsubo syndrome

All the patients received an infusion of norepinephrine or dopamine, as a vasopressor support for the septic shock before the TTS was diagnosed and to maintain adequate blood pressure afterwards. The administered exogenous catecholamines may have contributed to the induction of TTS. Pulmonary edema was presented in 6 patients, and mechanical ventilation or high flow nasal cannula was needed in 4 patients. Cardiogenic shock with an apical ballooning type occurred in 3 patients. The Ca^2+^-sensitizer levosimendan was used in 6 patients. Follow-up echocardiography demonstrated a substantial recovery of the cardiac function in all patients. In this series of 7 patients, 5 patients were discharged 1–2 weeks later, 2 patients died from multiorgan failure, which was attributed to the recurrence and exacerbation of septic shock and TTS.

## Discussion

Takotsubo syndrome has also been described as the ‘apical ballooning syndrome’, ‘broken heart syndrome’ and ‘stress cardiomyopathy’ in the literature. TTS shares some similar features with ACS. However, there are substantial differences between the two. TTS is now recognized as a separate clinical entity, which is characterized by transient regional wall motion abnormalities (RWMAs) of the left (and sometimes right) ventricle, which often result in a “circumferential pattern” of the left ventricular myocardial dysfunction, usually extending beyond a single epicardial vascular perfusion territory [5]. Based on the distribution of RWMAs, four different types of TTS have been described. The typical type, also known as the apical ballooning type, occurs in most patients (80%). Atypical forms include the midventricular pattern (15%), inverted (reverse) or basal pattern (2–5%) and focal pattern (1.5%) [2, 6, 7]. The biventricular pattern (right ventricular involvement) has also been increasingly reported [8, 9]. Data from the largest International Takotsubo Registry (n = 1750), conducted by Templin et al. [10], have revealed that approximately two-thirds of the patients have identifiable triggering events. Furthermore, although TTS is known as the broken heart syndrome, the prevalence of physical triggers exceeds that of emotional stress factors, which were predominant triggers in previous studies [2]. This was further confirmed by a 2019 systematic review of all available case reports by Nyman et al. [11], which corroborated that the substantial portion of TTS are preceded by various physical stressors, especially medical illness. With the growing knowledge about this syndrome and improved access to point-of-care echocardiography in the ICU, TTS is being increasingly observed in critically ill patients. Few studies on TTS in the ICU population have reported sepsis as a frequent triggering event [12, 13].

Our case series presented different findings on the septic shock-induced TTS. Regarding age, the mean age was 58 years, which is younger than the default age for emotional or other non-sepsis physical stress-induced TTS. The female predominance in other TTS populations was lost in this study. The predominant symptom of non-sepsis-induced TTS patients was chest pain, followed by dyspnea and syncope. The main presenting symptoms were acute respiratory and hemodynamic worsening, probably due to pulmonary oedema and severe impairment of the myocardial function. However, it is difficult to confirm whether pulmonary oedema was secondary to TTS or sepsis-associated acute respiratory distress syndrome. Furthermore, it is likely that all the patients with typical TTS developed cardiogenic shock via pump failure due to a larger area of the stunned myocardium. The atypical forms were not unusual in our series. Moreover, we reported a rare event of biventricular TTS in patient 7 who had an RV dysfunction in addition to the invert pattern of LV, while patient 6 presented a focal TTS. We are the first to describe these 2 infrequent patterns in patients with septic shock-induced TTS.

TTS diagnosis in patients with septic shock is challenging, since ECG changes are too similar to differentiate TTS from ACS. While a slight increase in the peak serum level of troponin combined with a substantially increased BNP or NT-proBNP can be used as a diagnostic clue, imaging is essential to make the diagnosis. TTE is the first-line imaging technique to identify the classical apical ballooning type, other circumferential patterns of RWMAs distribution and RV involvement [14]. In most cases of suspected TTS, invasive CAG and left ventriculography are frequently performed as it is diagnostic for TTS. CAG is crucial to exclude coronary arterial occlusions and left ventriculography can assess LV contractility, characteristic wall motion abnormalities with a non-coronary artery distribution showing and the variant morphologic patterns [1]. However, urgent CAG and left ventriculography in patients with septic shock are often unfeasible or are logistically challenging because of the life-threatening condition and deteriorating renal function. To this end, a joint consensus document of the European Association of Cardiovascular Imaging (EACVI) and the Japanese Society of Echocardiography (JSE) in 2020 recommended non-invasive CCTA as a potentially appropriate alternative to CAG in septic patients [15]. MCE can reveal whether the pronounced perfusion defect is present and improve the visualization of RWMAs in patients with a poor acoustic window. Hence, this bedside technique may be more promising for the early detection of TTS in septic shock patients. The diagnosis of focal TTS is much more difficult. LGE-CMR is always required to exclude acute myocardial infarction and myocarditis [1, 5, 15]. For patients who could not undergo CAG, CCTA, MCE or LGE-CMR, a marked discrepancy between the transient circumferential RWMAs and troponin level is used as the hallmark of TTS, indicating the absence of epicardial coronary artery obstruction [1, 15].

Sepsis-induced cardiomyopathy or septic cardiomyopathy (SCM) is the reversible left ventricular or biventricular depression and ventricular dilatation in patients with sepsis and septic shock, which has often been described for 40 years now [16]. In a recent review, Beesley et al. [17] reported the prevalence of septic cardiomyopathy to vary from 18 to 60% in septic shock patients depending on disparate definitions. However, the vast majority of studies in this field have focused on the parameters that assess the global LV performance, such as the stroke volume, ejection fraction and global longitudinal strain. Meanwhile, data on left ventricular RWMAs remain scarce. In an earlier study of 35 patients with septic shock, Ellrodt et al. [18] observed reversible segmental wall motion abnormalities of the left ventricle in 63% of the patients. More recently, Vallabhajosyula et al. [19] reported biventricular dysfunction in 114 (29%) of 388 patients with severe sepsis and septic shock. In the work of Vieillard-Baron et al. [20] right ventricular failure was reported to be frequently presented in 120 (42%) of 282 septic shock patients. Interestingly, in a large recent registry of TTS patients (n = 839), El-Battrawy et al. [21] identified an incidence of RV involvement of 11%. It seems that there are some common characteristics shared between sepsis-induced myocardial dysfunction and TTS. In addition, Autopsy studies in patients who died from septic shock showed an interstitial inflammatory infiltration in conjunction with contraction band necrosis, myocytolysis, interstitial fibrosis and interstitial oedema in the myocardium; some of these are classic histopathological findings in patients with TTS triggered by emotional or other physical stress factors [16]. Thus, there may exist an overlap between SCM and TTS.

Sepsis is a physical stressful condition which may trigger TTS. A recent systematic review by Cappelletti et al. [22] summarized 26 separate case reports of sepsis-triggered TTS. Another study by Lee et al. [23]. described 56 patients with TTS diagnosed by echocardiography, in which sepsis was the major (26.8%) trigger factor. In a Japanese database of 3719 eligible patients diagnosed with TTS, Isogai et al. [24] reported that sepsis was documented in 2.8% of the cases. A retrospective study conducted by Yerasi et al. [25] showed that sepsis was discovered in 16% of 345 TTS patients. In a study including 24,701 patients with TTS derived from the National Inpatient Samples (2008–2009, United States), El-Sayed et al. [26] found that sepsis was documented in 7.1% of the patients and increased the risk of developing TTS. Recently, the largest retrospective cohort study using the National Inpatient Samples database (2007–2013, United States) of all adults with severe sepsis by Vallabhajosyula et al. [27] revealed that TTS was identified in 10,746 admissions, accounting for 0.15% of all severe sepsis admissions, with a progressive increase in the frequency of TTS. These findings support the fact that a proportion of patients with SCM might suffer from TTS, especially those with segmental left ventricular dysfunction. Nevertheless, to date, we do not have enough data on the epidemiology of TTS in septic shock patients. In our ICU, over a period of 8 months, among 84 patients were admitted with septic shock, among which 7 cases of TTS (8.3%) were identified according to the new InterTAK Diagnostic Criteria. This relatively significant incidence of TTS in septic shock patients may be attributed to the expansion of echocardiography into critical care medicine and the increasing awareness among critical care physicians.

Although the exact pathophysiological mechanisms of TTS are still incompletely understood, it is widely accepted is that TTS is caused by the acute catecholamine surge from either an enhanced sympathetic stimulation or the administration of catecholamines.^2^ High levels of catecholamines might induce direct myocardial injury, negative inotropic effects by switching the β_2_-adrenoceptor from Gs to Gi coupling together with the desensitization of β_1_-adrenoceptor, and ischemic effects secondary to a coronary microvascular dysfunction [28, 29]. This model could also be used to explain the septic shock-induced TTS. Septic shock can cause sympathetic hyperactivity [3]. Besides, exogenous infusion of norepinephrine is the preferred first-line vasopressor in septic shock [30], which further increases the catecholamine levels in plasma. To date, there have been no randomized trials to define the optimal treatment of acute heart failure and cardiogenic shock in TTS. Therapeutic strategies are therefore based on clinical experience and expert consensus. Because of the central role of excessive adrenergic stimulation in the pathophysiology, once TTS is detected in septic shock patient, epinephrine infusion should be avoided, other vasopressor drugs (i.e., metaraminol or vasopressin) may be added to decrease norepinephrine dose, non-catecholamine inotropes (i.e., levosimendan or milrinone) may be a better choice [1, 5, 31].

## Limitations

The major limitation in our report is the small number of patients. The main reason for underdiagnosing TTS is the insufficient physician’s unawareness of this clinical entity. In addition, imaging modalities including CAG, CCTA and LGE-CMR are difficult to be performed in septic shock patients. Thus, we may have missed some patients with TTS. Larger studies are necessary to overcome these limitations. Furthermore, can non-catecholamine vasopressors help prevent TTS occurrence in septic shock patients? Are non-catecholamine inotropes and/or vasopressors or mechanical assist preferable in patients with septic shock-triggered TTS? Additional research needs to be conducted to answer these important questions.

## Conclusions

Takotsubo syndrome is not uncommon in septic shock patients and may be the true cause of a subset of patients with sepsis-induced cardiomyopathy. Echocardiography is the cornerstone of TTS diagnosis in patients with septic shock. Myocardial contrast perfusion echocardiography is more appropriate in these patients to rule out ACS. Considering the liberal use of catecholamine in septic shock patients, detection of TTS patients in this setting may be of particular importance.

## Data Availability

All data generated or analyzed during this study are contained within the present manuscript.

## Abbreviations

ACS: Acute coronary syndrome
BNP: Brain natriuretic peptide
CAG: Coronary angiography
CCTA: Coronary computed tomography angiography
CMR: Cardiac magnetic resonance
ECG: Electrocardiogram
ICU: Intensive care unit
LAD: Left anterior descending artery
LGE: Late gadolinium-enhancement
LV: Left ventricular
MCE: Myocardial contrast echocardiography
NT-proBNP: N-terminal pro-brain natriuretic peptide
RV: Right ventricular
RWMAs: Regional wall motion abnormalities
SCM: Septic cardiomyopathy
TTE: Transthoracic echocardiography
TTS: Takotsubo syndrome
